# Effects of colored lights on an individual's affective impressions in the observation process

**DOI:** 10.3389/fpsyg.2022.938636

**Published:** 2022-12-01

**Authors:** Xing Xie, Jun Cai, Hai Fang, Xiaoying Tang, Toshimasa Yamanaka

**Affiliations:** ^1^School of Art and Design, Guangdong University of Technology, Guangzhou, China; ^2^Academy of Arts and Design, Tsinghua University, Beijing, China; ^3^Faculty of Art and Design, Tsukuba University, Tsukuba, Japan

**Keywords:** color, lighting, mood, impressions, emotional experience

## Abstract

The lighting environment has an important influence on the psychological and physical aspects of a person. On certain occasions, reasonable lighting design can regulate people's emotions and improve their feelings of comfort in a space. Besides, specific lighting can create a specific atmosphere according to space requirements. However, in the study of an individual's affective impressions, there is still some uncertainty about how colored lights affect an individual's moods and impressions toward visual objects. This research improves the understanding of the emotional impact of colored light in space. To better understand the lighting environment in the observation process, the project studied the effects of four groups of lights (green, blue, red, and yellow) on the participants' moods and impressions. Participants watched two sets of visual images under four different lighting conditions and provided feedback on their emotions and evaluations through the Multiple Mood States Scale, Two-Dimensional Mood Scale, and Semantic Differential Scale. The results show that different colors of light have a significant effect on mood, and red light can arouse emotional changes to calm, irritated, relaxed, nervous, stability, and pleasure. At the same time, different colors of light have a certain relevance to participants' impressions and this provides further research value for the design of the colored light environment in an individual's affective impressions. Therefore, this study discusses the feasibility of colored lights as a display method, which has potential application prospects for constructing different space atmospheres.

## Introduction

The process of observing a visual object occurs in various display spaces in our daily life, such as people observing graphics, images, and objects that can be seen everywhere, as well as observing scenes in movies and games (Castelhano and Krzyś, 2020). This kind of display space is not limited to museums and art galleries, and is found even in shopping centers, homes, laboratories, or entertainment spaces. Moreover, with the development of human–computer interactions, the metaverse, and other technologies into all aspects of our lives, display space involves not only the observation of these visual objects in the real space environment, but also the virtual environment and even the combination of them (Song and Yamada, 2019). An important part of this observation process is an individual's experience in the space. An individual's experience is influenced by various sensory factors. Research suggests that an individual's experience will be affected by factors such as labels, the way of hanging/display, and illumination (Pelowski et al., 2017). The visual effect of illumination is an important factor to be taken into account in the selection of lighting in a space, which can guide visual attention and provide color and rendering scenarios for the environment (David et al., 2019). The artistic expression of lighting is inherently diverse, and the fusion between it and the expression of visual objects is an interesting part of art communication (Schielke, 2020). Recently, with the development of digital technology, colored lights have been widely used to improve the emotional experience of individuals in space (Lee and Lee, 2022). As we value the storytelling and immersive experience of display space, it is particularly important to understand the role of design elements in the space, especially illumination since this is considered to be one of the most complex design elements that combines technology, perception, and appreciation (Kuijsters et al., 2015).

The design of illumination in space has a certain universality, but it is unique due to the different interrelationships among lighting, people, visual objects, and space needs (Lee, 2019). Therefore, to provide a high-quality emotional experience as the display space needs, an abstract study of lighting, individuals, visual objects, and space atmosphere is particularly important.

A common field of research related to lighting and display space is museum research. Museum exhibitions have stricter requirements for lighting to avoid damage to artworks (Hurlbert and Cuttle, 2020). Art exhibitions are more innovative in lighting applications, creating better visual experiences. Choosing the illumination of visual objects is generally determined by designers or curators, and they are of a personal nature in many cases (Pridmore, 2017; Pelowski et al., 2019). Another more empirical criterion is that visual preferences can be expressed by observers based on strictly controlled experiments (Nascimento and Masuda, 2014). Early research on the preferences of visual objects was primarily based on daylight illumination, because natural daylight, incandescent spotlights, or a combination of both was commonly used in exhibitions and was considered to be the best suitable lighting for color rendering (Pinto et al., 2008; Smet et al., 2011). Recently, with the development and popularization of energy-efficient solid-state lighting sources, researchers have considered other sources, such as LEDs (light-emitting diodes), and have discussed many aspects. In terms of the feasibility of white LED lighting, by the design of triband white-light LED lighting, similar luminous efficacies with similar products can be achieved in a continuous spectrum that does not emit UV (ultraviolet) or IR (infrared radiation), protecting artworks (Berns, 2011). As far as the best LED lighting parameters are concerned, research shows that “visibility” and “warmth” (texture) are two factors of perception toward paintings; for the main factor of “visibility,” observers prefer the correlated color temperature (CCT) to be at about 3500K (Zhai et al., 2016). Research on the stability of pigments indicates that certain colorants are more vulnerable to degradation when exposed to LED lighting (Richardson et al., 2020). In addition, research on visitors' emotional experience shows that upon performing actual tests under gallery conditions, changing the lighting will not make a noticeable difference when there is an opportunity to personally choose the lighting; the average CCT chosen is 3777K (Pelowski et al., 2019). Since the invention and innovation of different light sources have a major impact on people's lifestyles and behaviors, research on the influence of colored lights on people's mood has also received increasing attention (Kurt and Osueke, 2014; McDonald et al., 2022). The results show that colored lights can make an entire space more comfortable and relaxing in many specific environments, such as the home (Figueiro et al., 2015), office (Hubalek et al., 2010; Figueiro and Rea, 2016), and hospital (Zraati, 2012); emotions will further affect people's perception, cognition, coping, and creativity (Izard, 1993). From the theoretical perspective of light research, de Kort (2019) summarized the theoretical structure of pathways of light relevant to psychological functioning, with the aim of informing future lighting research. At the same time, she emphasized the importance of various light effects and the significance of choosing the best lighting design parameters. Therefore, the research on various light effects in the display space, especially the research on colored lights, needs to be supplemented.

How individuals can understand visual objects is a core topic in display space (Kim and Lee, 2016). On the one hand, the color of visual objects can have a significant impact on human expression and emotions, for example, comparing white flowers and red flowers; viewing yellow flowers is more relaxing (Xie et al., 2021). In the display space, dark green and red plants are more suitable for children's areas to improve the energy of visitors, and green-yellow and bright green plants are more likely to attract attention (Elsadek et al., 2017). On the other hand, as Hurlbert and Cuttle (2020) mentioned, there should be a richer understanding of how light affects an individual's impressions of seeing and feeling, and how to use lighting to enhance the expression of visual objects to enrich their emotional experience. An individual's impressions of visual objects, including attention, perception, appreciation, and imagination, can be a creative process and can be interpreted as an independent entity that is separated from the designer (Leder et al., 2004). Using light intervention, an individual's impressions may be affected in many ways. Different lighting arrangements, light source colors, and CCTs can be used to enhance the impressions of the entire space, such as the clarity, spaciousness, relaxation, privacy, pleasantness, and order (Flynn et al., 1973; Flynn and Spencer, 1977; Durak et al., 2007; Li et al., 2021). Also, lighting can affect people's impressions of paintings. The CCT will affect an individual's overall appreciation of the arrangement, whereas the overall hue of paintings and the background lightness have little effect (Feltrin et al., 2020). Studies have also discussed the interaction between the colors of visual objects and lighting. For example, participants were invited to view impressionist paintings under different CCT lighting conditions. The main colors of these paintings were red, blue, green, and yellow. The experiment evaluated the individual's impressions of the paintings' colors, vividness, brightness, attractiveness, background color, and arrangement. The results show that, regardless of the main color tone of the artwork, a series of preference trends indicate that the perceived warmth and brightness decrease with an increase in the CCT (Feltrin et al., 2017). Besides, studies have pointed out that an individual's attention to visual objects will depend on whether they have an artistic background (O'hare, 1976). In addition, with the popularity of colored indoor lighting, the impact of people observing visual objects in a colored light environment needs to be further studied. Zraati (2012) focused on the psychological and physical effects of color and mentioned that in the follow-up research, the relationships between objects and colors in the waiting area will be explored. Sokolova and Fernández-Caballero (2015) discussed the combination of light and color for effective computing, and proposed that people of different ages and countries may respond differently when exposed to combinations of light and color. However, there is still not enough evidence to prove the relationship between colored lights and an individual's impressions toward visual objects, and whether colored lights can arouse different moods and impressions requires more in-depth research.

The major purpose of this study is the exploration of more display methods and values as a means to awaken an individual's affective and cognitive states in a display space. In particular, previous studies did not attempt to jointly measure the relationships between colored light, mood, and impressions. It should be meaningful to use colored light as a display method in the sense of constructing different space atmospheres.

## Conceptual framework and hypotheses development

**Figure 1:**
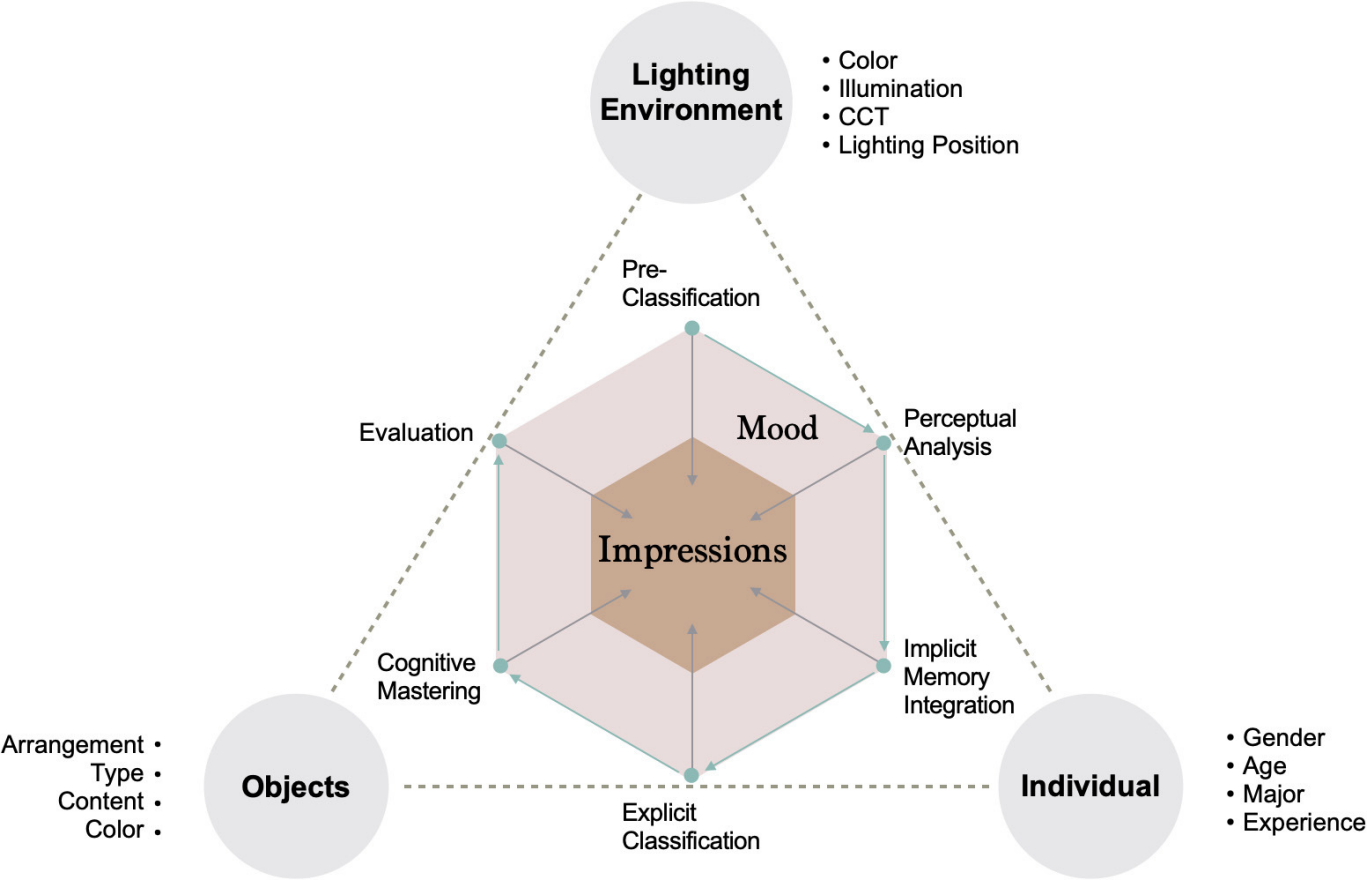
Conceptual model of the experiment (Leder et al., 2004; Leder and Nadal, 2014; Pelowski et al., 2017; Mastandrea et al., 2021).

To discuss the relationship between colored light and individuals, we adopted a Model of Art Processing (Leder et al., 2004; Leder and Nadal, 2014; Pelowski et al., 2017; Mastandrea et al., 2021) to consider the specific content of the experimental design. This model is widely used for the empirical study of an individual's aesthetic experience, which is a process of the affective and cognitive states generated through a series of information processing stages (Leder et al., 2004). As shown in Figure 1, a series of information processing stages are proposed, which include pre-classification, perceptual analysis, implicit memory integration, explicit classification, cognitive mastering, and evaluation. In this work, we focus on factors for the lighting environment, objects, and visitors that influence the processing stages in a specific experimental design. The specific experimental controls are as follows: for the lighting environment factor, control the color of light, illumination, CCT, and lighting position; for the visual objects factor, control of the arrangements, type, content, and color; for the individual factor, control of the gender, age, major, and experience. At the same time, we believe that the potential impact of mood and impression factors on the processing stages could be different: (1) pre-classification, during which the mood of the individuals will be affected by the pre-existing mood state, and the impressions toward the artwork which will be affected by the most intuitive visual factors (e.g., arrangement, style, and color); (2) perceptual analysis, which includes first impressions that are influenced by simple features (e.g., color, clarity, and simple content), and mood generated by the influence of light and visual objects; (3) implicit memory integration, which is affected by personal experiences (including the mood state of memory and the contrast or explanation of past experiences) of moods and impressions; (4) explicit classification, which involves modifying mood through deep feelings (e.g., beauty, fun, and more details); (5) cognitive mastering, based on independent perspectives generated by personal emotional fusion combined with specific types of mood as well as impressions from different levels of understanding and imagination; (6) evaluation, meaning that individuals will evaluate their emotional and cognitive states in some way, and will stop processing information when they are satisfied. This is reflected in regulation and changes in mood at this stage, as well as personal expression of the meaning of the artwork. In general, the stimulation of these factors will awaken the information processing of implicit memory and explicit knowledge, and will thus produce an interpretation of emotion and cognition. Therefore, it is important to understand what kind of moods and impressions are evoked and, through evaluation, to understand the mechanisms of aesthetic experience.

To study the effects of colored light on the individual's moods and impressions, we designed two experiments, using the same laboratory environment and participant selection methods. In this laboratory setting, four colored light conditions (green, blue, red, and yellow) and two types of visual objects (realistic and abstract images) were regulated. In terms of measurement methods, considering that the emotional state is a dynamic changing process, we used two kinds of emotional scales to obtain emotional state data. The first experiment used a Multiple Mood States Scale (Terasaki et al., 1992), which can reflect the current mood state; the second experiment used a Two-Dimensional Mood Scale (TDMS) (Sakairi et al., 2013), which defines the momentary mood state through comparison of pre- and post-intervention. The purpose was to find a better way to judge the mood state in visual processing under different lighting conditions. To determine consistent impression patterns among people in the display space upon changing the color of light, different dependent variables of impressions were measured using the Semantic Differential Scale (Flynn and Spencer, 1977).

We believe that the combination of different types of images and colored light may produce different results in individuals' moods and impressions. Our hypotheses are as follows: H1; the control of colored light can adjust the mood and impression of individuals, H2; under the influence of colored light, the individual's mood will have a certain correlation with their impressions, particularly the impact of mood on an interesting impression, H3; under the influence of colored light, abstract images more easily arouse the individual's impressions than realistic images.

## Experiment 1: Method

### Laboratory setup

**Figure 2:**
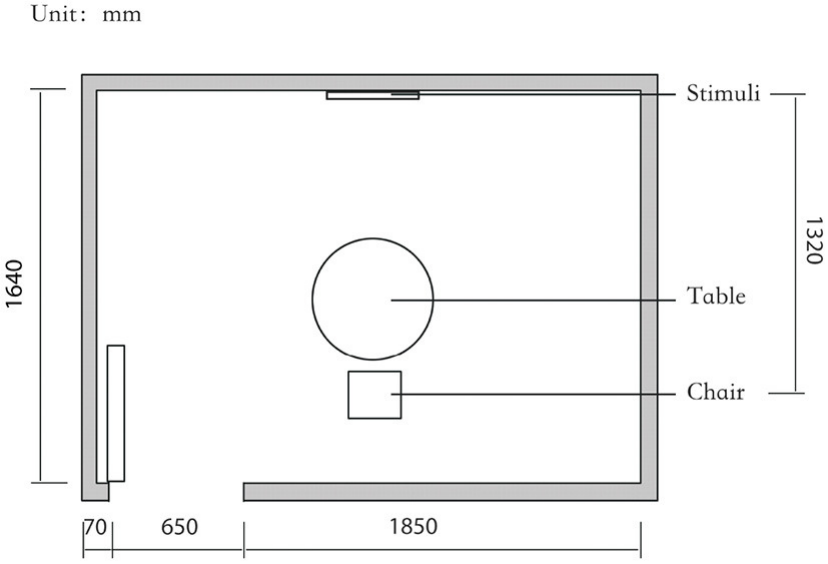
Laboratory simulation space plan.

To strictly control the effect of experimental light on the subjects, an experimental darkroom was selected as the simulation space (see Figure 2). The room size was 2.5 x 1.64 x 2 m, with no influence from natural light, and the relative humidity was maintained at 30% during the experiment. The temperature was constant at 24°C. There is a certain difference between RGB color and light color. Therefore, this research comprehensively considered the color gamut, saturation, control technology, and other factors, and we finally selected four colors in the experiment: green, blue, red, and yellow. The light color calibration is shown in Table 1; the related data were measured using a Spectroradiometer JETI Specbos 1201. The light equipment used was from a Philips hue white and color ambiance starter kit A60, and the illuminance on the picture surface was controlled at 250 lx. One lighting position was above the picture on a white wall, and the other was at the center of the ceiling in order to render the entire space. The waiting area for resting and completing information forms was outside the darkroom. The lighting condition of the waiting room was 5700k and 500lx.

**Table 1:** Light color calibration.

**Lighting color**	**Spectrogram**	**Color coordinates (x, y)**
Green		0.289, 0.478
	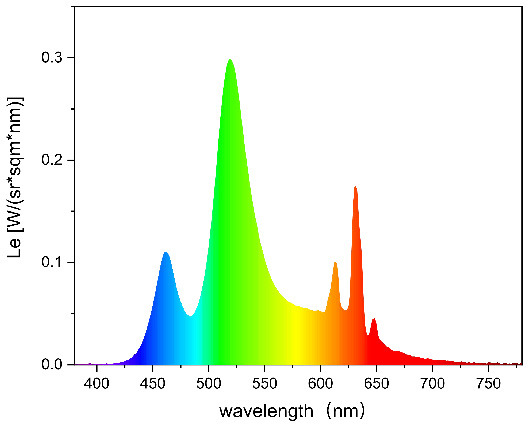	
		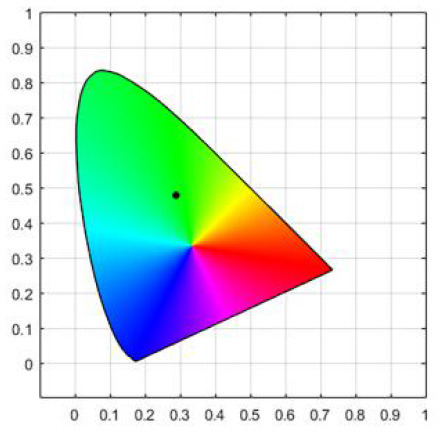
Blue		0.191, 0.127
	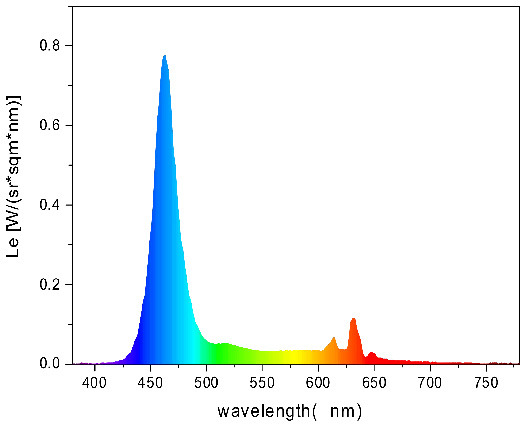	
		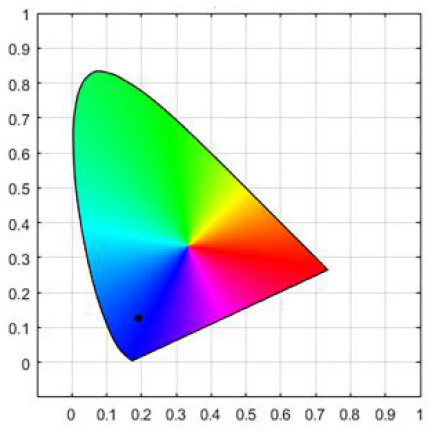
Red		0.562, 0.368
	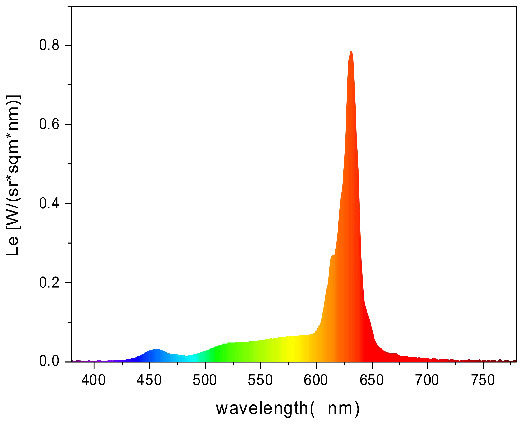	
		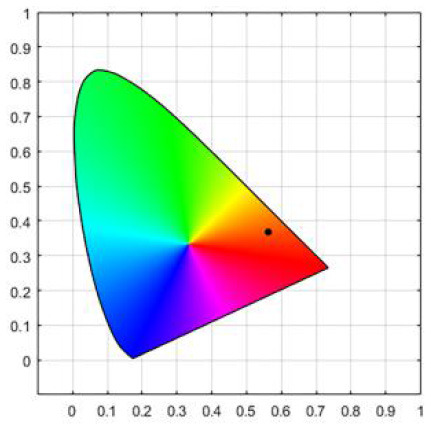
Yellow		0.443, 0.465
	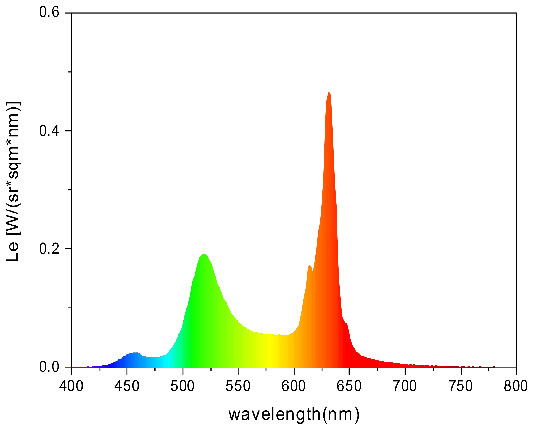	
		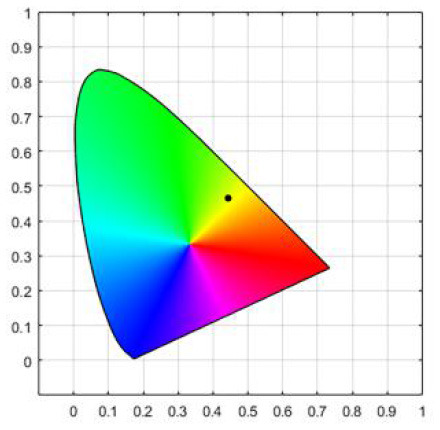

### Stimuli

To control the effects of the stimulus on color perception, black and white pictures were used as experimental materials. The display method was A3 sized prints which were hung on the wall of the experimental darkroom. To prevent reflection of the light affecting the visual perception of the subjects, matte photo paper was used.

**Figure 3:**
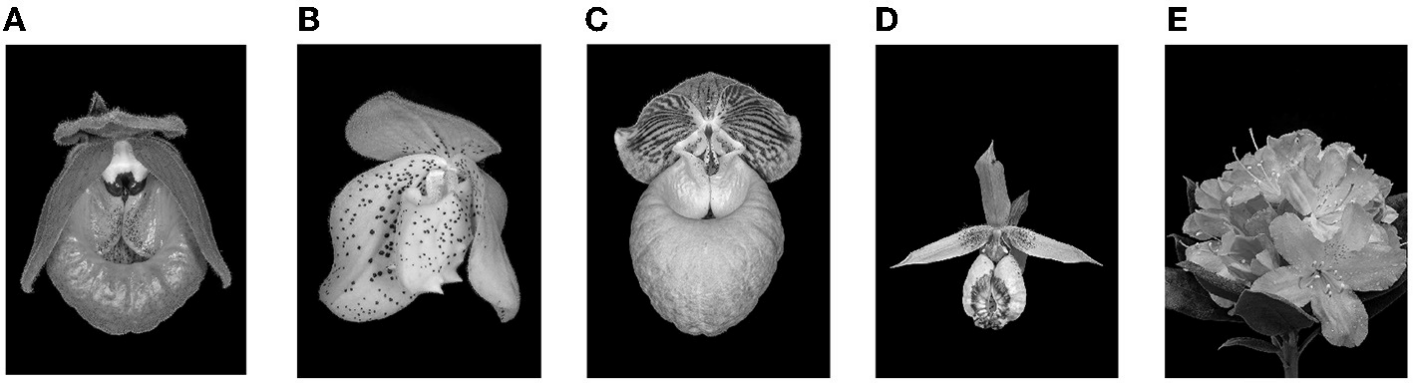
Experimental materials: flowers **(A–E)**.

Twelve Chinese students from a university in Japan participated as volunteers through open recruitment in the pre-experimental material selection (six male students, six female students, ages 20–30, five art and design majors, and seven non-art and design major). In the test, five pictures (see Figure 3, copyright from nature photographer Xin Zhong) were selected from 12 black and white flower photography works. The selection criterion was low familiarity, and the accuracy of guessing the original image color was centered. In the formal experiment, a within-subject design method was adopted, and each participant saw four different colors of light on five pictures. To reduce sampling and measurement errors and to avoid additional variability due to the order of pictures, the five pictures were randomly ordered during the experiment.

### Questionnaire

The experimental questionnaire consisted of three parts. The first part was basic information, including gender, age, and specialty. This part was helpful to classifiy and analyze later data. The second part was a nine-item question extracted from the Multiple Mood States Scale (see Table 2) to measure the mood state, from “1: not at all” to “5: totally agree.” The third part was the Semantic Differential Scale (see Table 3). Subjects scored 12 opposite pairs of factors, describing their subjective impressions of the experimental materials in different lighting conditions. The selection of these factors was based on scales used in previous research to understand visitors' impressions toward visual objects (Zhai et al., 2015; Bhattacharjee and Pal, 2019).

**Table 2:** Multiple Mood States Scale.

**9 questions of Multiple Mood States Scale (M1-M9)**			
M1	I feel excited.	M6	I feel lonely.
M2	I feel nervous.	M7	I like this atmosphere.
M3	I feel my mind is clear.	M8	I am willing to talk to others.
M4	I feel that it is difficult to concentrate.	M9	I think other people will like it.
M5	I feel depressed.		

**Table 3:** Semantic Differential Scale.

**12 pairs of words in the Semantic Differential Scale (SD1-SD12)**			
SD1	Dark - Bright	SD7	Ugly - Beautiful
SD2	Cold - Warm	SD8	Gloomy - Cheerful
SD3	Blurry - Clear	SD9	Weak - Strong
SD4	Glaring - Soft	SD10	Boring - Interesting
SD5	Dull - Rich	SD11	Unnatural - Natural
SD6	Static - Dynamic	SD12	Pessimistic - Optimistic

### Participants

Through open recruitment, eight Chinese observers aged 20–30 participated in the experiment as volunteers, including four male observers and four female observers. They were all students from a university in Japan; four professionals in art and design and four non-art and design-related majors. Before the experiment, the informed consent form was signed by all observers. All observers used a color vision tester Panel D-15 to check the judgment of color. Those who were completely correct participated in formal experiments. The participants provided written informed consent and received 500 JPY at the end of the study. The study was conducted in accordance with the Declaration of Helsinki, and the protocol was approved by the Ethics Committee of the first author's institution.

### Procedure

**Figure 4:**
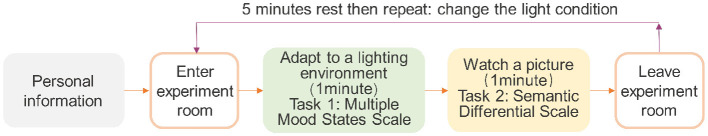
Procedure for experiment 1.

As shown in Figure 4, observers entered the waiting area, completed basic information and informed consent, and then started the experiment after learning the relevant experimental instructions and completing the color vision check. (1) Observers entered the experimental dark room with one type of lighting condition. After 1 min of adaptation, they evaluated the emotion affected by colored light using the Multiple Mood States Scale. (2) Then, they observed five black and white pictures. After 1 min, they evaluated the pictures one at a time using the Semantic Differential Scale. (3) Observers left the experimental dark room and waited for 5 min in the rest area, during which the testers changed the lighting conditions. (4) Steps (1) to (3) were repeated. Observers were allowed to adapt to the next lighting condition. The lighting and picture display sequences were random for each observer.

## Experiment 1: Results and discussion

### Colored light and mood

**Figure 5:**
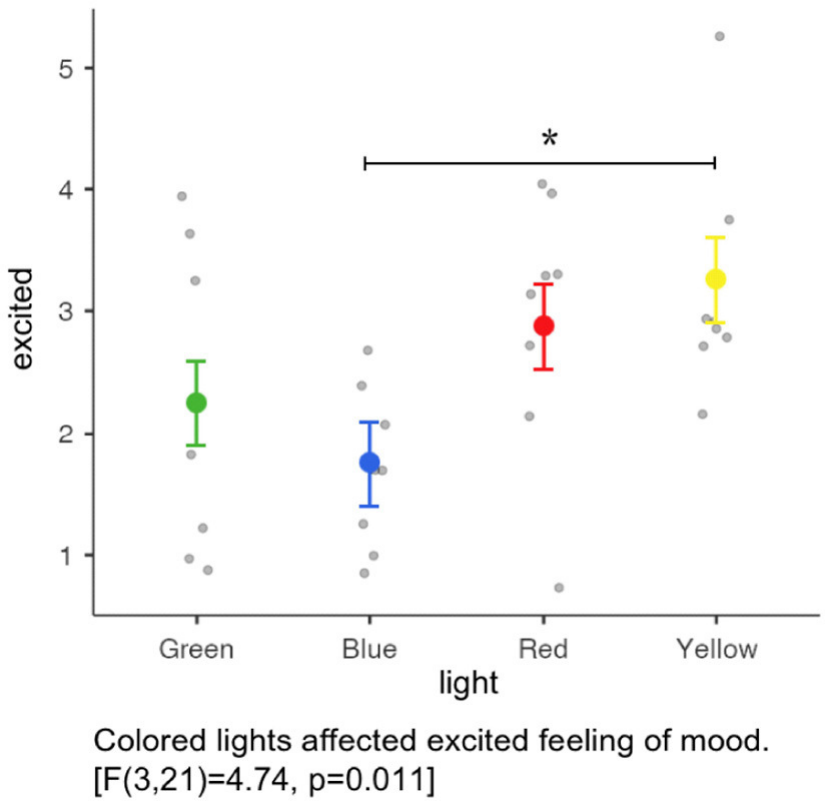
Result of participants' excited mood in experiment 1. *p < 0.05.

Data were analyzed using SPSS 25.0 and Jamovi. To determine the mood, a repeated measures ANOVA method was used. Different colors of light had a significant effect on the subject's excited mood [F_(3, 21)_ = 4.74, *p* = 0.011] (see Figure 5). There was a significant difference between yellow light and blue light [p = 0.011]. The results for feelings of nervous, mind is clear, difficult to concentrate, depressed, lonely, like this atmosphere, willing to talk to others, and guess other people will like it, did not exhibit significant differences.

### Colored lights and impressions

**Figure 6:**
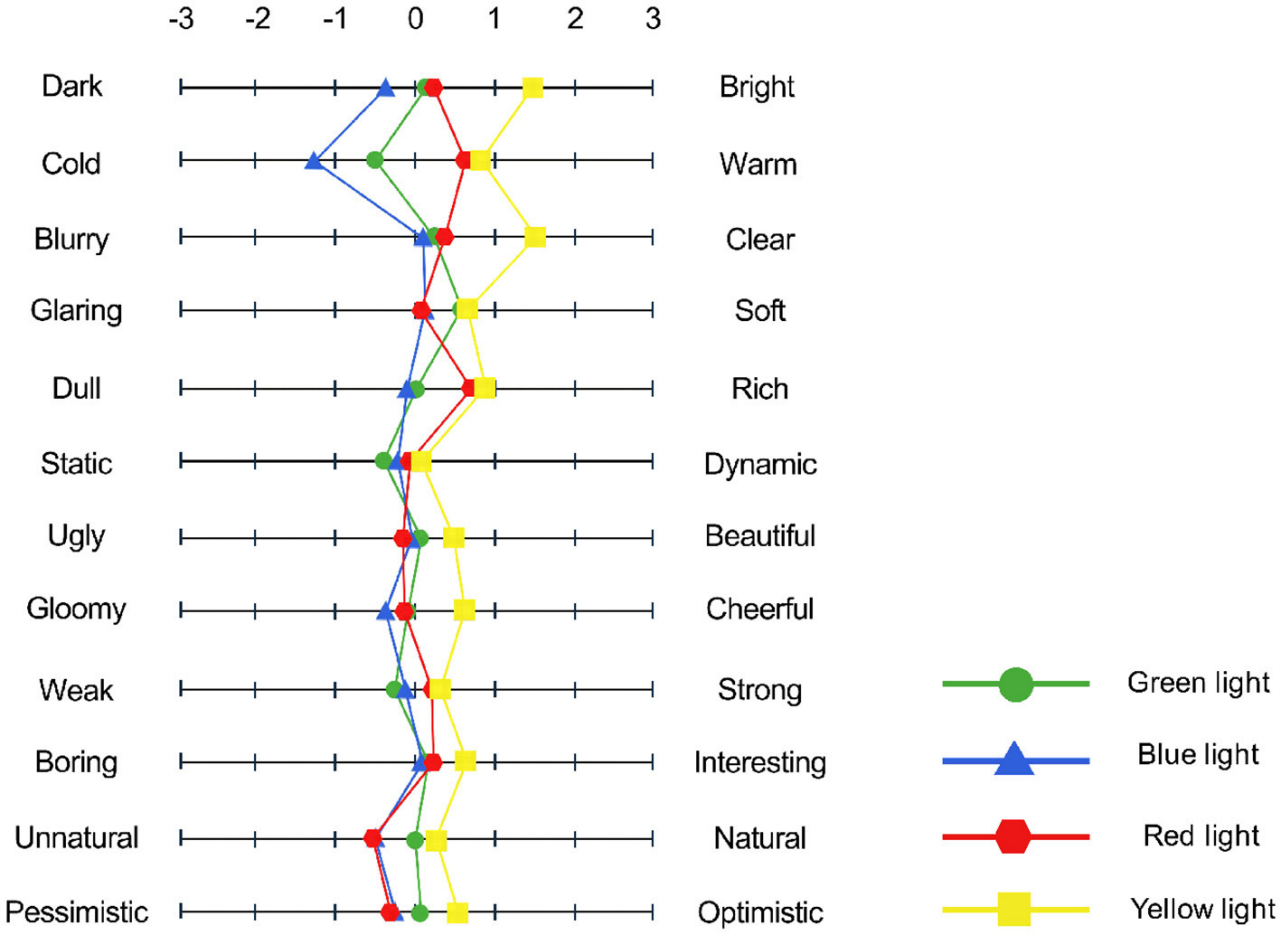
Degree of Semantic Differential Scale.

As shown in Figure 6, the preliminary results of the Semantic Differential Scale under different lighting conditions show that the scores for all words under yellow light conditions were higher than the other three light conditions. Under the blue light condition, the scores for dark-bright, cold-warm, blurry-clear, dull-rich, gloomy-cheerful, and boring-interesting were lowest. As a result, yellow light is the most suitable light for daily use and visual work, and it can have a positive effect on the overall visual impression.

**Figure 7:**
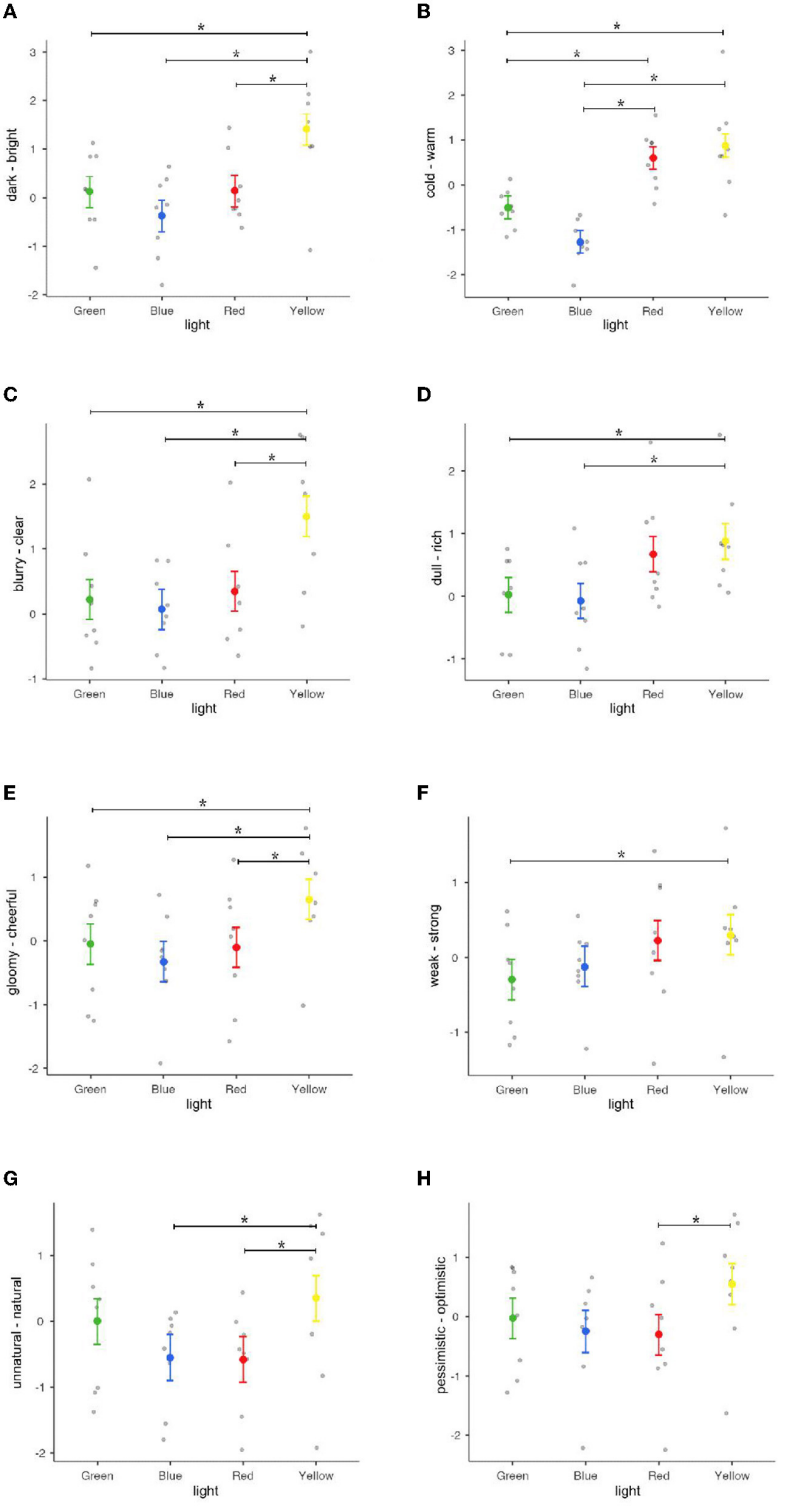
Result of participants' impressions in experiment 1: **(A)** Dark-bright impression, **(B)** cold-warm impression, **(C)** blurry-clear impression, **(D)** dull-rich impression, **(E)** gloomy-cheerful impression, **(F)** weak-strong impression, **(G)** unnatural-natural impression, and **(H)** pessimistic-optimistic impression. *p < 0.05.

The pictures themselves did not significantly affect the impressions of the participants. A repeated measures ANOVA method was performed based on the scores of the 12 groups of words in the Semantic Differential Scale. The results show that different colors of light have a certain effect on the impressions toward pictures (see Figure 7), especially the dark-bright impression [F_(3, 21)_ = 8.35, *p* = < 0.001), cold-warm impression [F_(3, 21)_ = 18.2, *p* = < 0.001], blurry-clear impression [F_(3, 21)_ = 5.62, *p* = 0.005], dull-rich impression [F_(3, 21)_ = 4.01, *p* = 0.021], gloomy-cheerful impression [F_(3, 21)_ = 6.71, *p* = 0.002], weak-strong impression [F_(3, 21)_ = 3.78, *p* = 0.026], unnatural-natural impression [F_(3, 21)_ = 4.64, *p* = 0.012], and pessimistic-optimistic impression [F_(3, 21)_ = 3.79, *p* = 0.026].

Besides, majors presented significant results for the boring-interesting impression [F_(1, 158)_ = 4.768, *p* = 0.030] and the pessimistic-optimistic impression [F_(1, 158)_ = 6.547, *p* = 0.011]. Participants with art and design backgrounds had more interesting and optimistic impressions, but for other vocabularies, there was no significant difference between different professional backgrounds.

### Moods and impressions

Table 4 shows the three factors identified by the factor analysis carried out in the Multiple Mood States Scale. Factor 1, related to “sensory,” exhibited a total variance of 45%, including nervous, difficult to concentrate, depressed, and lonely feeling. Factor 2, related to “preference,” presented a total variance of 16%, including mind is clear, like this atmosphere, and guess other people will like it. Factor 3, related to “sharing,” displayed a total variance of 14%, including excited and willing to talk to others. In particular, it is worth mentioning that a stronger feeling of excited corresponded to a willingness to communicate.

**Table 4:** Rotated component matrix of mood factor analysis.

	**Factor 1 sensory (45%)**	**Factor 2** **preference (16%)**	**Factor 3 sharing (14%)**
Excited	−0.317	0.464	0.675
Nervous	0.772	−0.165	−0.216
Mind is clear	0.064	0.908	−0.129
Difficult to concentrate	0.457	−0.307	0.287
Depressed	0.814	−0.110	0.015
Lonely	0.899	−0.009	−0.170
Like this atmosphere	−0.612	0.679	0.056
Willing to talk to others	0.000	−0.146	0.870
Other people may like it	−0.470	0.669	0.417

**Figure 8:**
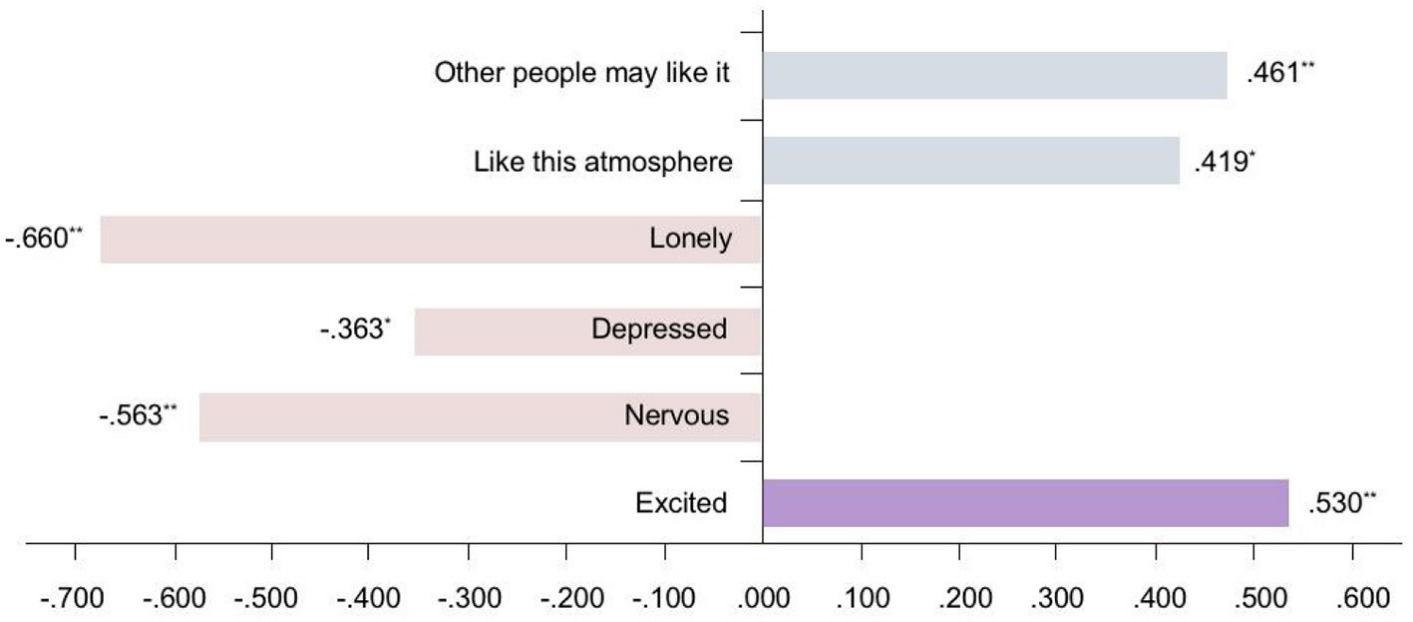
Spearman correlation coefficients between the boring-interesting impression and mood.

In terms of a correlation between the Multiple Mood States Scale and the Semantic Differential Scale, Figure 8 shows the Spearman correlation coefficients between the boring-interesting impression and mood (words of mood that are not related to boring-interesting impressions are not listed). It can be seen that the lonely mood presented the highest negative correlation coefficient, and the excited mood exhibited the highest positive correlation coefficient. The keywords in factor 1 that were related to “sensory” were all negative in mood. Therefore, the moods of lonely, depressed, and nervous are all negatively related to the impression of interest.

### Discussion

The results show that proper colored light can play a positive role in regulating individuals' mood. The four colors of light produce significant differences in the excited mood, of which yellow and blue light exhibit differences. As noted in previous studies, blue light has a calming effect on people (Viola et al., 2008). Therefore, regarding the design intent of a space atmosphere, blue light can be distinguished from daily used light (yellow light).

The bright impression induced by yellow light is different from the other three colors of light; all scores of impressions are positive results. This is probably because people prefer the color yellow, which is close to natural light, and yellow light is the most comfortable of the four lights for processing visual information (Wan et al., 2012). Different colors of light illuminate the pictures, and impressions of cold and warm are the most obvious. There is no difference between red and yellow lights; both can produce a warm feeling and bring out a warm atmosphere. Green and blue tend to be cold, especially blue light.

It can be seen from the factor analysis that the main factor “sensory” is a significant part of the emotional state, followed by “preferences” and “sharing.” These factors are closely related to visitors' interest in visual objects. Therefore, in the practice of space design, the interest of visual and other objects in the space can be enhanced through the design of lighting scenes.

Besides, the findings in experiment 1 suggest that professional background may affect specific impressions toward realistic images. According to previous studies, researchers believed that people who are not in the arts and design professions may focus on the realism of the artworks (O'hare, 1976), while art and design background visitors will be more concerned about the style and visual effects of artworks (Cupchik and Gebotys, 1988). This study shows that participants with an art and design background have a strong interest and positive impressions toward the combination of colored light and visual objects. The results show that colored lights that affect impressions toward visual objects are irrelevant to the medium of pictures considered in this study. Therefore, it can be said that these findings can be applied to the display of realistic images of flowers. However, it is not enough to study realistic images, and abstract images still require further exploration.

## Experiment 2: Method

Based on experiment 1, we further explored the effect of colored light on participants' moods and impressions toward abstract images. We thus designed experiment 2 and improved the procedures, including changing the experimental materials and questionnaires. The reason for this is that the realistic nature of flower photography has certain limitations on the perspectives of imagination and artistry. At the same time, the participants observed the four different colors of light-matched works, which caused the experiment to run too long and the pictures to be viewed too much. Moreover, complicated questions on the Multiple Mood State Scale caused a certain amount of pressure on participants. Therefore, experiment 2 used the same laboratory space as experiment 1 with details of the procedures redesigned for the above considerations.

### Stimuli

**Figure 9:**
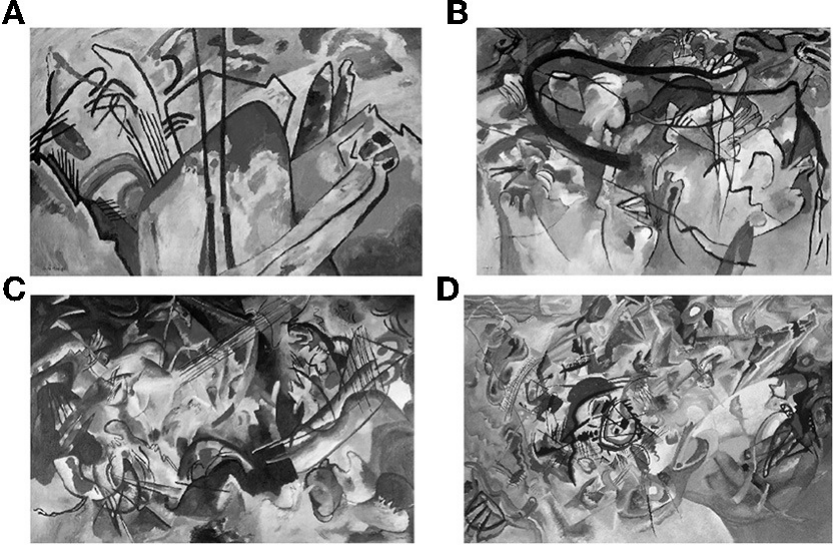
Experimental materials. Kandinsky's paintings: **(A)** Composition IV, 1911, **(B)** composition V, 1911, **(C)** composition VI, 1913, and **(D)** composition VII, 1913.

Kandinsky's paintings have been used as experimental materials due to his unique abstract thinking and imagination (Tanaka and Matsumoto, 2013). In this experiment, 16 Chinese students from a university in Japan participated as volunteers through open recruitment in the material selection pre-experiment (eight male students, eight female students, ages 20 to 30, eight arts and design majors, and eight non-arts and design), and finally selected four paintings with low familiarities and strong correlations, which are Kandinsky's four series of paintings (see Figure 9), named composition IV, 1911; composition V, 1911; composition VI, 1913; and composition VII, 1913. The black and white display method was identical to experiment 1. Using the within-subject design in the experiment, each participant observed four different colors of the light-matched unique pictures, and the order of presentation of the stimulus and matching with light conditions were randomized.

### Questionnaire

**Figure 10:**
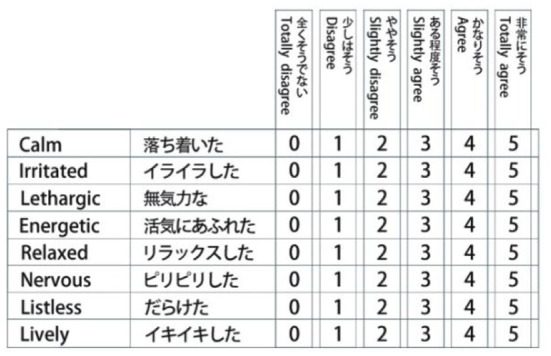
Two-Dimensional Mood Scale (TDMS) in English and Japanese.

The experimental questionnaire consisted of four parts. The first part was basic information, which was identical to experiment 1. The second part was the Two-Dimensional Mood Scale (TDMS), which measures the emotional state based on answers to eight questions. It analyzes the emotional state through changes in the scores of subjects before and after different light interventions (see Figure 10). The third part was the Semantic Differential Scale (see Table 5). Based on the results of the scale used in experiment 1, eight groups of adjectives were selected for experiment 2. The participants described the different groups of adjectives by scoring them in a different light, describing their subjective perception of the experimental materials in the different environments.

**Table 5:** Semantic Differential Scale.

Eight pairs of words in the Semantic Differential Scale (SD1-SD8)			
SD1	Dark - Bright	SD5	Ugly - Beautiful
SD2	Cold - Warm	SD6	Gloomy - Cheerful
SD3	Blurry - Clear	SD7	Boring - Interesting
SD4	Dull - Rich	SD8	Unimaginative-Imaginative

### Participant

Through open recruitment, 36 Chinese observers aged 20–30 participated in the experiment as volunteers. The final effective number was 32, including 14 male observers and 18 female observers. They were all students from a university in Japan; 12 professionals in art and design, and 20 non-art and design-related majors. Before the experiment, the informed consent form was signed by the observers. All observers used the color vision tester Panel D-15 to check their judgment of color. Those who were completely correct participated in the formal experiments. The participants provided written informed consent and received 500 JPY at the end of the study. The study was conducted in accordance with the Declaration of Helsinki, and the protocol was approved by the Ethics Committee of the first author's institution.

### Procedure

**Figure 11:**
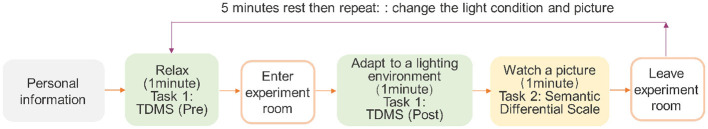
Procedure for experiment 2.

As shown in Figure 11, observers entered the waiting area, completed basic information and informed consent, and then started the experiment after learning the relevant experimental instructions and completing the color vision check. (1) Observers completed a (pre) TDMS in the waiting area to obtain mood data before being affected by colored light. (2) Observers entered the experimental dark room with one type of lighting condition present. After 1 min of adaptation, they evaluated the emotion affected by colored light using a (post) TDMS. (3) They observed Kandinsky's pictures for 1 min and completed the Semantic Differential Scale. (4) Observers left the experimental dark room and waited for 5 min in the waiting area, during which the testers changed the lighting and pictures. (5) Steps (1) to (4) were repeated. Observers were allowed to adapt to the next lighting condition. The lighting and picture display sequences were random for each observer.

## Experiment 2: Results and discussion

### Colored lights and mood

In terms of emotions, the eight questions in the TDMS were: calm (a), irritated (b), lethargic (c), energetic (d), nervous (e), nervous (f), listless (g), and lively (h). Using the following formulas, we obtained four results for vitality (V), stability (S), pleasure (P), and arousal (A):

Vitality: V = d + h – c – g
Stability: S = a + e – b – f
Pleasure: P = V + S
Arousal: A = V – S

A total of 12 results were tested using two related samples of non-parametric tests in order to determine differences in the following four conditions: red light intervention, blue light intervention, green light intervention, and yellow light intervention. The results of the TDMS before and after the interventions are shown in Table 6. Under different colored light conditions, some items presented significant results in the emotional sense. Red light displayed significant differences in the moods calm (*p* = 0.010), irritated (*p* = 0.012), relaxed (*p* = 0.012), nervous (*p* = 0.008), stability (*p* = 0.001), and pleasure (*p* = 0.003). Under the condition of blue light, the participants presented significant differences in the moods irritated (*p* = 0.011), relaxed (*p* = 0.044), and stability (*p* = 0.024). Under the conditions of green and yellow light, there were significant differences in the moods pleasure (*p* = 0.025) and irritated (*p* = 0.027).

**Table 6:** Changes in Two-Dimensional Mood Scale scores pre- and post-intervention.

		**Red Light**			**Blue Light**			**Green Light**			**Yellow Light**		
		**Pre**	**Post**	**↑or ↓**	**Pre**	**Post**	**↑or ↓**	**Pre**	**Post**	**↑or ↓**	**Pre**	**Post**	**↑or ↓**
a	M	3.44	2.97	↓^**^ p = 0.010	3.41	3.09		3.53	3.34		3.41	3.28	
	SD	1.045	1.177		1.160	0.995		1.077	0.971		0.946	1.114	
b	M	0.66	1.09	↑^*^ p = 0.012	0.56	0.94	↑^*^p = 0.011	0.72	0.63		0.72	0.91	↓^*^p = 0.027
	SD	0.745	0.995		0.840	0.878		1.114	0.707		0.888	0.995	
e	M	3.37	2.691	↓^*^ p = 0.012	3.44	3.00	↓^*^p = 0.044	3.31	3.00		3.50	2.97	
	SD	1.157	1.424		0.878	1.191		1.091	1.191		0.880	1.231	
f	M	0.66	1.22	↑^**^ p = 0.008	0.66	0.87		0.50	0.78		0.69	0.84	
	SD	0.865	1.157		0.745	0.751		0.762	0.792		0.965	0.954	
S	M	5.50	3.34	↓^**^ p = 0.001	5.62	4.28	↓^*^p = 0.024	5.63	4.94		5.50	4.50	
	SD	2.423	3.376		2.166	2.808		2.624	2.139		2.436	2.874	
P	M	8.06	5.34	↓^**^ p = 0.003	8.03	6.34		8.41	7.06	↓^*^ p = 0.025	8.19	6.94	
	SD	5.162	6.073		4.652	5.976		4.354	4.362		4.967	6.430	

^*^p < 0.05.

^**^p < 0.01.

### Colored lights and impressions

In terms of impressions, the Semantic Differential Scale was used to measure participants' impressions under different lighting conditions. Through multivariate analysis of variance (MANOVA), the following conclusions can be drawn (see Table 7):

1. The two factors light color and pictures, significantly impacted participants' impressions, with significances of 0.049 and 0.000, respectively; however, the interaction effect was not significant (*p* = 0.303), indicating that the color of light and the pictures significantly affected the participant. There was no synergy in the impact of the evaluation of pictures.
2. Different colors of light exhibited significant differences in the cold-warm impression of the participants [F_(3, 112)_ = 3.504, *p* = 0.018], and the four pictures presented no significant difference in the cold-warm impression (*p* = 0.753). The interaction of the colored lights and pictures exhibited a significant difference in the cold-warm impression [F_(9, 112)_ = 2.392, *p* = 0.016, Wilk's Λ = 0.161].
3. The effects of colored lights presented no significant differences in the dark-bright (*p* = 0.577), dull-rich (*p* = 0.056), and fuzzy-clear (*p* = 0.716) impressions. However, the effects of pictures exhibited significant differences in the impressions of dark-bright [F_(3, 112)_ = 4.521, *p* = 0.005], dull-rich [F_(3, 112)_ = 6.456, *p* = 0.000], and blurry-clear [F_(3, 112)_ = 5.268, *p* = 0.002].

**Table 7:** Probability (P) value of MANOVA.

**Set of SD**	**Factor 1**	**Factor 2**	**Interaction**
	**Light**	**Picture**	**Light** **×Picture**
Dark - Bright	0.557	0.005	0.213
Cold - Warm	0.018	0.753	0.016
Blurry - Clear	0.719	0.002	0.842
Dull - Rich	0.056	0.000	0.231
Ugly - Beautiful	0.531	0.255	0.203
Gloomy - Cheerful	0.279	0.497	0.332
Boring - Interesting	0.435	0.141	0.391
Unimaginative-imaginative	0.458	0.122	0.599

Besides, majors presented significant results for the dull-rich impression [F_(1, 126)_ = 4.148, *p* = 0.044]. Although the results of experiment 2 show that interesting and optimistic impressions did not differ significantly between different professional backgrounds, the related impressions for participants with art and design backgrounds were still higher than those with non-art and design backgrounds participants.

### Discussion

In this experiment, the TDMS measures the change of mood state before and after the light interventions; we can obtain results before and after the use of colored light, which more intuitively reflects the process of emotional change. These results indicate that red light has the most significant effect on mood changes, followed by blue light. Green light reduces the feeling of pleasure, and yellow light relieves irritated feelings, that is, for yellow light, as a common form of daily light, the impact of mood change is weak. Previous research showed that participants experienced higher levels of tension, anger, depression, and anxiety and lower levels of visual comfort, attractiveness, brightness, and calmness of environment in red environments compared with white, for both cold and warm light (Shahidi et al., 2021). According to different space requirements, the design and application of colored lights should be more targeted, such as in medical and office spaces, the purpose of which is to reduce people's anxiety, which would make the use of yellow light more suitable. For the purpose of creating different emotional experience spaces, it is necessary to choose a suitable colored light according to the scenario and theme (Kuijsters et al., 2015).

Colored lights only affect the cold-warm impression, and Kandinsky's pictures factor into the impressions of dark-bright, dull-rich, and blurry-clear. This is different from our initial hypothesis. In the selection of experimental materials, we originally believed that four pictures belonging to the same type of materials would not produce any major differences; however, in the actual emotional experience, they have significantly influenced individuals. Previous research has shown that the novelty and complexity of pictures have a certain potential to arouse the observers' response (Martindale et al., 1990). This proves that the rich visual effects of the abstract images themselves can play an important role in the aesthetic processing of individuals' impressions, and have indeed been playing an important role in aesthetic studies (Iigaya et al., 2021).

## General discussion

### The value of methods for measuring light and emotional experience

Just like other design elements in the space, different colors of light have a rich impact on the mood state of the emotional experience. In a complex space environment, this study strictly controlled the experimental environment and materials, introduced the Multiple Mood States Scale and TDMS to measure the mood state caused by colored lights, and reflected on the differences between the two scales. The Multiple Mood States Scale can reflect the current state of mood, while the TDMS defines the momentary mood state through comparison pre- and post-intervention. The value of TDMS is in the measurement of mood states, and from the perspective of measurement effectiveness, we can focus on momentary mood states rather than continual mood. Especially in experience research, how to enhance and create different experiences requires understanding of the relationship between colored lights and mood changes. A comparison with similar research topics shows that the experimental results obtained here for the effects of colored light on individuals' mood are reliable. It can be seen that the experimental method, reflecting a specific population in a specific space, can be applied to the study of light and mood, and to some extent, it can draw objective and credible conclusions.

Besides, the semantic differential method as well as free comments can be used to obtain individuals' impressions. The successful use of the Semantic Differential Scale in this experiment once again verified the applicability of the self-reporting method to experiments involving light and impressions.

### Colored lighting design for positive and negative mood

The mood state in aesthetic experience is dynamic and diverse. Although we think that the aesthetic experience itself is usually positive (pleasant and interesting), previous research has shown that it can also lead to negative (unpleasant and uninteresting) results. In particular, the possibility of negative results in laboratory tests is even greater (Leder et al., 2004). This may be because, in the laboratory, the participants deal with many types of stimuli in a certain time and space, and provide exact answers, whereas in a real environment, individuals can freely choose the place and visual objects according to their interests. Moreover, in real display space, mood state is difficult to measure directly. Our research also shows some negative mood state results. For example, red and blue light increases the sense of irritation and reduces the moods relaxation and stability; red light can also reduce the moods calm and pleasure and increase the mood nervousness.

To understand how colored lighting regulates mood, scholars have explained the meaning of positive and negative mood. Positive mood increases social and expansive tendencies (Cunningham, 1988). Previous research discussed the overall design approach using mood state as the driving force, and proposed to build imaginable and meaningful relationships between people and objects to stimulate any of the 25 positive emotions, including inspiration, hope, joy, satisfaction, relaxation, and dreaminess (Desmet, 2012). A negative mood will reduce our willingness to participate, but it can prompt people to sit down and think, to be alone, and to be calm (Desmet, 2015). It is indeed possible to intentionally use a negative mood to create a rich experience (Fokkinga and Desmet, 2013). In different space design purposes, colored lights can be used to render a specific atmosphere to stimulate people's emotional experiences. The artist Kohei Nawa's art exhibit “Foam” (Nawa, 2019) reminds the visitors to calmly think about the origin of life while applying blue light to render the atmosphere. The theme of the exhibition and the design of the lights are in line with our research results. Whether the result of a positive or negative mood state, this research provides valuable design opportunities for aesthetic experience and colored lighting design.

### Colored lighting design in display space

In the study of impressions toward visual objects, the results of experiment 1 showed that different colors of light have an impact on the impressions of flower pictures, and the flower pictures themselves do not affect the participants' impressions; however, in experiment 2, different colors of light only affect the cold-warm impression, and Kandinsky's paintings affect the impressions of dark-bright, dull-rich, and blurry-clear. The difference between realistic and abstract images may be the cause of this result, and also may due to the limitation of insufficient number of participants in the study, further improvements are needed in further in-depth experimental research in the future. Previous studies have found that stimulus materials can affect the participant's response to pictures. One important factors is the picture's degree of realism, and another factor that cannot be ignored is the clarity of the picture (Berlyne, 1971; O'hare, 1976). This suggests that, despite utilizing the same series of works by the same artist, under the influence of real lighting conditions and the properties of visual objects, individuals can still generate different impressions. Therefore, in display design, the distinction between different visual objects should be further explored.

In display space, one of the ways to draw individuals' attention to the visual objects, the environment, and even the content of the space to generate further perception, appreciation, and imagination, is to arouse interest, which can make information easier and more effective to identify. Our study demonstrates the connection between individuals' interest and mood. Positive moods such as excited, like this atmosphere, and guessing that others' affection for it, are shown to be related to a positive interest impression. Although no more specific results were obtained in this study, there are indications that the state of mood and impression, caused by lighting as an environmental factor, has a certain connection with the awakening of interest, and may thus attract further research attention. These findings inspire new considerations regarding individuals' perception of lighting in display space.

## Conclusion

Compared with white lighting or even natural lighting, this study focuses on individuals' affective impression responses produced by observing objects in a colored, illuminated space. By comparing four typical colors of light (green, blue, red, and yellow), we observe that red light reduces feelings of calm, relaxed, stability, and pleasure, and elevates feelings of irritated and nervous. Blue light reduces feelings of relaxed and stability and increases irritated feelings. Green light reduces the feeling of pleasure and yellow light reduces irritated feelings. At the same time, this study shows that the interaction between the type of artwork and the lighting has an impact on the perception of visual objects. When observing a realistic image, the individual's impressions are only affected by the colored lights, whereas when observing an abstract image, the image itself and the colored lights jointly affect the individual's impressions. Therefore, for the design of emotional experiences through control and organization of colored lights and visual objects suggests that colored light may contribute to rich affective changes and cognitive responses.

These discoveries may affect the design of display spaces and may become a means to stimulate individuals' attention, perception, appreciation, and imagination of the space and visual objects. It is worth mentioning that this study has some limitations, such as a laboratory space setting instead of a real display space. Therefore, it is necessary to conduct further study under real display space conditions to confirm the above findings, and to study the characteristics and lighting effects of different types of display space. as well as under more specific colored light display conditions, such as dynamic lighting and interactive media, it is worthwhile to conduct other studies to evaluate the combination of colored lights, the frequency of light changes, and the time of change, etc. In addition, since the representativeness of this study needs to be further enriched, this provides a direction for further in-depth research of our experiments.

To this end, we must continue to explore the different ways in which lighting stimulates individuals' emotional experiences.

## Data availability statement

The raw data supporting the conclusions of this article will be made available by the authors, without undue reservation.

## Ethics statement

The studies involving human participants were reviewed and approved by the Academic Ethics Committee of Guangdong University of Technology. The patients/participants provided their written informed consent to participate in this study.

## Author contributions

XX, TY, HF, and XT substantial contributions to the conception. XX and TY designed this work. XX conducted experiments, analyzed data, and wrote the manuscript. TY, HF, and JC revised and edited the manuscript. All authors contributed to the article and approved the submitted version.

## Conflict of interest

The authors declare that the research was conducted in the absence of any commercial or financial relationships that could be construed as a potential conflict of interest.

## Publisher's note

All claims expressed in this article are solely those of the authors and do not necessarily represent those of their affiliated organizations, or those of the publisher, the editors and the reviewers. Any product that may be evaluated in this article, or claim that may be made by its manufacturer, is not guaranteed or endorsed by the publisher.
